# National Infectious Diseases Surveillance data of South Korea

**DOI:** 10.4178/epih/e2014030

**Published:** 2014-11-11

**Authors:** Sunhee Park, Eunhee Cho

**Affiliations:** Division of Infectious Disease Surveillance, Korea Centers for Disease Control and Prevention, Osong, Korea

**Keywords:** National notifiable diseases, Infectious disease, Surveillance data, Mandatory surveillance system, Sentinel surveillance system

## Abstract

The Korea Centers for Disease Control and Prevention (KCDC) operate infectious disease surveillance systems to monitor national disease incidence. Since 1954, Korea has collected data on various infectious diseases in accordance with the Infectious Disease Control and Prevention Act. All physicians (including those working in Oriental medicine) who diagnose a patient with an infectious disease or conduct a postmortem examination of an infectious disease case are obliged to report the disease to the system. These reported data are incorporated into the database of the National Infectious Disease Surveillance System, which has been providing web-based real-time surveillance data on infectious diseases since 2001. In addition, the KCDC analyzes reported data and publishes the Infectious Disease Surveillance Yearbook annually.

## INTRODUCTION

In general, national notifiable infectious diseases are those that cause severe infections with poor prognoses; are highly contagious and can be prevented with appropriate quarantine, vaccination, or chemical therapy; or require management and control by conducting research. The designation of a notifiable disease is multi factorial. As a result, there are numerous discrepancies in the definition of these diseases across various countries. The incidence of infectious diseases and issues related to these diseases change with time; therefore, frequent updates to relevant information are required. The purpose of designating national notifiable infectious diseases is to protect the public from the threat of infectious diseases by effectively controlling the disease using an accurate and sensitive surveillance system and by appropriately allocating the available resources.

The incidence of national notifiable infectious diseases is calculated using data reported by medical providers (Western and Oriental medical doctors) to health institutions in accordance with the Infectious Disease Control and Prevention Act [1]. Physicians working at clinics or hospitals report cases of infectious disease to their regional health center. These data are reviewed by local health authorities and transferred to the Korea Centers for Disease Control and Prevention (KCDC). After final confirmation from the KCDC, a report of these national statistics is published. According to the diagnosis and reporting standards in the “case definitions for national notifiable infectious diseases [2],” cases with an infectious disease are classified as three groups; an infectious disease patient (pathogen identifier), a patient with a suspected infection (unidentified pathogen carrier), or a pathogen carrier. The scope of each infectious disease report varies depending on the disease characteristics.

The Mandatory Surveillance System requires obligatory reporting on infectious diseases ‘without delay’ to a district health center. In comparison, the Sentinel Surveillance System receives the report on weekly from the designated sites. In addition, the Complementary Sentinel Surveillance System consists of a school-based infectious disease surveillance program and ocular infectious disease surveillance program.

Since the start of the surveillance system, many changes to the computer system structure have made. In the early stages of the construction of the surveillance system, a client-server based method between health institutions was the main way of data collection. However, from January 18, 2007, the reporting method using paper-based assessment was converted to a computer-based assessment. This computerized disease surveillance system was more efficient, flexible, and convenient than the paper-based assessment. In 2009, an internet-based reporting system was developed for use in clinics and hospitals. This system allowed for near real-time assessments the incidence of infectious diseases nationwide as well as quick data collection and a rapid response. From May 18, 2007, a web statistics system on infectious disease (http://stat.cdc.go.kr) was established. These efforts improved public accessibility to system data. In 2011, this website was updated as the infectious disease web-statistics system (http://is.cdc.go.kr/nstat/index.jsp) and it provides data on 54 infectious diseases that are mandatorily reported. In addition, the Infectious Disease Surveillance Yearbook, which organizes and analyzes the overall incidence of national notifiable infectious diseases, is published and distributed annually [3]. Reported infectious disease surveillance data since 1954 which is based on the Infectious Disease Control and Prevention Act is presented in Table 1.

## DATA ACCESSIBILITY

The Disease Web Statistics System (http://is.cdc.go.kr/nstat/index.jsp) provides real-time data on 54 national notifiable infectious diseases specified by area, sex, age, infected region, and period. Moreover, it is possible to compare the current weekly incidence to that of the previous year. These data can be presented as tables or figures, and downloaded as a spreadsheet.

## KEY FINDINGS AND PUBLICATIONS

The Sentinel Surveillance System monitors 24 of the 78 national notifiable infectious diseases. The KCDC uses these data to estimate the trends and patterns of some infectious diseases. The data only consists of the number of reported cases at each sentinel site and does not contain detailed information of each individual case; therefore, the national incidence of sentinel infectious diseases is difficult to estimate using the sentinel surveillance data. The main purpose of this data is to determine trends and patterns of infectious diseases.

**Figure 1.**
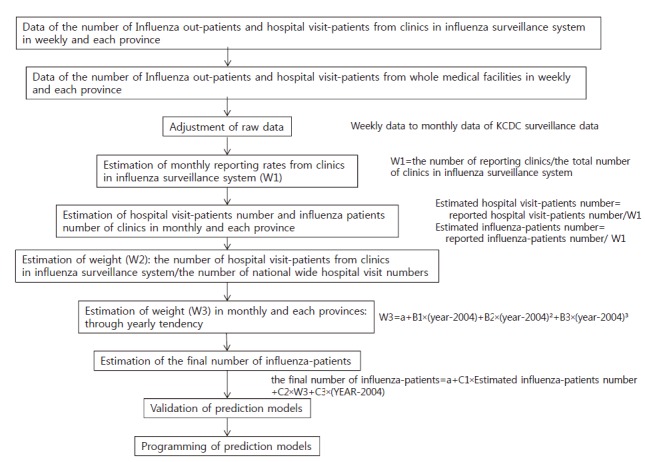
Procedure of model development to calculate influenza incidence. KCDC, Korea Centers for Disease Control and Prevention; a is constant, B1, B2, B3, C1, C2, and C3 are coefficients from this regression model. From Roh YK. Modeling the national pandemic influenza cases and inpatients of pneumonia in Korea. Osong: Korea Centers for Disease Control and Prevention; 2011 [4].

For example, one study used these sentinel surveillance data to analyze influenza cases and hospitalizations of severe pneumonia nationwide [4]. This analysis used the sentinel surveillance data on sentinel influenza sites from 2005 to 2010 as well as national health insurance claims data from the Health Insurance Review and Assessment Service (influenza diagnostic codes J09-J11). In addition, the number of influenza patients per month, the total numbers of out-patients, and the total numbers of hospital visits were extracted for analysis. With these data, an estimation model was created (Figure 1) [4].

In this paper, the number of influenza patients in Seoul on November 2009 was estimated using this formula; final number of patients=constant + C1×(estimated number of patients ×W3)+C2×(year-2004). Minor differences between the estimated value based on the model and the health insurance claims data were found.

## STRENGTHS AND WEAKNESSES

The reporting of national notifiable diseases is standardized and provides up-to-date information including diagnostics and the epidemiological characteristics of each infectious disease. Moreover, these data are distributed to healthcare sites regularly. However, the reporting of national notifiable infectious diseases in South Korea relies on the passive reporting of healthcare workers. Thus, the reporting rate is relatively low. Nevertheless, the number of patients with an infectious disease can be estimated using existing data. The KCDC not only seeks to encourage the reporting of infectious diseases that present at healthcare sites but also continues to develop ways to improve the current systems using multidimensional approaches, not limited to passive reporting.

## Figures and Tables

**Table 1.** Reported cases of national infectious diseases in South Korea, 1954-2013

Disease	1954-1959	1960-1969	1970-1979	1980-1989	1990-1999	2000-2009	2010-2013	Total
Cholera	0	1,972	206	145	196	210	14	2,743
Typhoid fever	5,398	40,790	13,018	2,481	3,012	2,198	566	67,463
Paratyphoid fever	193	440	64	172	164	795	223	2,051
Shigellosis	1,004	2,705	1,703	534	3,368	6,986	783	17,083
Enterohemorrhagic *Escherichia coli*	-	-	-	-	-	431	246	677
Viral hepatitis A	-	-	-	-	-	-	7,585	7,585
Pertussis	75,941	75,002	18,187	8,381	444	186	390	178,531
Tetanus	0	0	6	53	12	109	72	252
Measles	67,901	116,435	48,877	26,075	13,327	56,061	266	328,942
Mumps	25,314	32,945	15,186	10,483	13,084	28,099	36,747	161,858
Rubella	-	-	-	-	-	413	142	555
Japanese encephalitis	11,763	14,587	862	1,641	18	33	63	28,967
Varicella	-	-	-	-	-	81,291	125,773	207,064
Malaria	-	8,497	35,689	0	9,765	18,576	3,585	76,112
Hansen’s disease	-	1,891	10,952	2,881	1,166	423	25	17,338
Scarlet fever	19	37	170	1,808	1,210	977	5,158	9,360
Meningococcal meningitis	116	157	36	81	40	114	29	457
Legionellosis	-	-	-	-	-	106	104	210
*Vibrio vulnificus* sepsis	-	-	-	-	-	536	244	780
Murine typhus	-	0	4	4	136	378	137	659
Scrub typhus	-	-	-	-	3,534	42,761	29,791	76,086
Leptospirosis	-	-	-	596	432	1,193	193	2,414
Brucellosis	-	-	-	-	-	620	83	703
Rabies	-	268	28	2	1	5	0	304
Hemorrhagic fever with renal syndrome	-	-	349	450	1,230	3,683	1,734	7,446
Primary/secondary/congenital syphilis	-	-	-	-	-	-	2,551	2,551
Creutzfeldt-Jakob disease	-	-	-	-	-	-	108	108
Dengue fever	-	-	-	-	-	321	598	919
Q fever	-	-	-	-	-	51	42	93
West Nile fever	-	-	-	-	-	-	1	1
Lyme borreliosis	-	-	-	-	-	-	16	16
Melioidosis	-	-	-	-	-	-	3	3
Chikungunya fever	-	-	-	-	-	-	2	2
Sever fever with thrombocytopenia syndrome	-	-	-	-	-	-	36	36

From Korea Centers for Disease Control and Prevention (KCDC). 2013 Infectious diseases surveillance yearbook. Osong: KCDC; 2014 [3].
