# Supplementary material for: National Infectious Diseases Surveillance data of South Korea
Source: Epidemiol Health. 2014 Nov 11;36:e2014030. doi: 10.4178/epih/e2014030 (PMC4272235; doi:10.4178/epih/e2014030)
Supplement: Supplementary file 1 [file epih-36-e2014030-supplementaldata.pdf]

한국 법정감염병 자료

박선희[1,] 조은희[1]
질병관리본부 감염병감시과[1]

Korea National Notifiable Infectious Diseases Data

Park Sun-Hee[1]
Division of Infectious Disease Surveillance, Korea Centers for Disease Control & Prevention[1]

Correspondence
- Cho Eun-Hee, MD, PhD. Division of Infectious Disease Surveillance, Korea Centers For Disease Control & Prevention, 187, Osongseangmyeong2-ro, Osong-eup, Cheongwon-gun, Chungcheongbuk-do 363-951, Korea. Tel: +82-43-719-7160, Fax: +82-43-719-7188, Email: cho6404@korea.kr
- Received April 15, 2014; Accepted April 25, 2014

Summary

법정감염병 관리를 위해 가장 기본으로 질병발생을 파악하기 위하여 감염병의 신고를 기본으로 한 감염병 감시체계를 운영하고 있다. 우리나라는 1954년부터 '감염병의 예방 및 관리에 관한 법률'을 근간으로 감염병 통계를 산출하고 있으며, 감염병 신고의무는 환자를 진단 또는 사체를 검안한 의사(한의사)에게 있다. 신고자료는 국가 감염병감시 시스템의 데이터베이스로 운영되고 있어, 2001년부터 현재까지 실시간으로 감염병 발생 현황자료를 감염병웹통계로 공개하고 있다. 또한 매년 국가감염병감시시스템(National Infectious Disease Surveillance System, NIDDSS)을 통해 신고·보고된 법정감염병 발생현황을 분석하고 정리한 「감염병 감시연보」를 발간하여 배포하고 있다.

Key message

감염병웹통계(http://is.cdc.go.kr)에서는 전수감시 대상 54종 감염병에 대한 지역별, 성별, 연령별, 감염지역별, 기간별, 전년대비 주간 발생현황 등 실시간 기간 검색이 가능하며, 검색된 통계를 표 또는 다양한 그래프로 볼 수 있으며, 또한 이러한 자료들은 엑셀로 다운로드하여 사용이 가능하다. 또한 표본감시 대상 감염병 통계도 제공되고 있다.

Introduction

일반적으로 한 국가(혹은 지역)에서 임상적으로 중증 감염이고 예후가 나쁜 질환과 전염성이 강하고, 격리나 백신 혹은 화학요법 등에 의하여 타인에게 전파를 예방할 수 있는 경우, 또는 예방·치료법이나 그 효과의 연구를 위한 역학적 자료나 정보가 필요한 경우 등 예방관리가 필요한 경우 법정감염병으로 지정된다. 그러나 법정감염병으로 어떤 질병을 지정해야하는가라는 문제는 상당히 복합적이여서 국가적으로 큰 차이를 보이고 있다. 감염병 유형이 시간적으로 변하기 때문에 시간이 지나감에 따라 법정감염병의 내용이 달라지어 새로이 재검토되고 변화한다. 감염병 중 법정감염병이라고 법으로 정하는 목적은 국가가 관리함으로써 그 감염병으로부터 국민을 보호하며 한정된 국가자원의 효율적 배분으로 효과적인 관리를 위하여 감시체계 운영 및 관리 사업의 대상을 정확히 하기위하여 제공하는 것이다.

법정감염병의 발생자료는 「감염병의 예방 및 관리에 관한 법률」에 의거하여 감염병 환자 등을 진단한 의료인(의사, 한의사 등)이 보건기관에 신고한 자료를 기초로 하고 있다[1]. 병·의원 의사로부터 일차적으로 관할 보건소로 신고되는 감염병 환자 발생 신고 사례는 보건소 및 시·도의 검토를 거쳐 질병관리본부로 보고되고, 질병관리본부의 최종 승인을 통해 법정전염병환자의 집계가 이루어진다. 감염병 환자에 대한 구분은 법정감염병 진단·신고 기준에 따라 환자(병원체 확인자), 의사환자(병원체 미확인자), 병원체보유자로 구분하며, 질병의 특성에 따라 감염병별로 신고범위를 달리하고 있다[2]. 우리나라의 감염병 감시체계는 지난 2000년 전염병예방법의 개정을 통해 국가 감시대상 감염병의 목록과 신고체계를 대폭 개편함은 물론 감시 효율 제고를 위한 지속적인 시스템의 개선 등을 통해 많은 발전을 이루어왔다. 감염병 감시체계는 감염병 발생 시 의무적으로 '지체없이' 관할 보건소에 신고하도록 하는 전수감시체계(Mandatory Surveillance System), 일정한 기준에 의해 참여하는 의료기관을 표본감시 기관으로 지정하여 7일 이내에 관할 보건소/질병관리본부에 신고하도록 하는 표본감시체계(Sentinel Surveillance System), 이외 특정 감염병 발생현황 및 추이를 파악하고 법정감염병 감시체계의 미비점을 보완하는 학교감염병감시체계, 안과감염병감시체계 등 보완적 감시체계(Complementary Sentinel Surveillance System)이 있다. 시스템 측면에서도 많은 변화가 있었다. 감시시스템 구축 초기에는 감시기관간에 클라이언트-서버 방식으로 운영되어오던 것을 2007년 1월 18일부터 웹기반 운영체계로 전환하여 전염병 발생현황 파악이 종전의 서면집계 방식(paper-based)에서 전산화된 질병감시시스템(computerized disease surveillance system)으로 발전하면서 시스템 운용의 유연성, 사용자 편의성을 도모하는 측면에서 시스템의 효율성을 높였고, 2009년 병의원 인터넷 신고시스템을 개발하여 전국의 감염병 발생현황을 준실시간적(near real time)으로 파악할 수 있게 되었을 뿐만 아니라 신속한 대응조치 역시 가능하게 되었다. 또한 감염병 감시 자료의 효율적인 환류 일환으로 2007년 5월 18일부터는 「감염병웹통계시스템」(http://stat.cdc.go.kr)을 새롭게 구축·운영함으로써 누구나 쉽게 인터넷을 통해 감염병 감시자료를 이용

할 수 있도록 접근성을 제고하였고, 2011년 감염병 웹통계(http://is.cdc.go.kr)로 개편 하였다. 이 사이트에서는 전수감시대상 54종 감염병에 대하여 개인정보를 제외한 자료를 제공하고 있다. 또한 매년 국가감염병감시시스템(National Infectious Disease Surveillance System, NIDDSS)을 통해 신고·보고된 법정감염병 발생현황을 분석하고 정리한 「감염병 감시연보」를 발간하여 배포하고 있다[3].

표 1 감염병 신고현황

구분	질병명	1954-1959	1960-1969	1970-1979	1980-1989	1990-1999	2000-2009	2010-2013	합 계
제1군	콜레라	0	1,972	206	145	196	210	14	2,743
	장티푸스	5,398	40,790	13,018	2,481	3,012	2,198	566	67,463
	파라티푸스	193	440	64	172	164	795	223	2,051
	세균성이질	1,004	2,705	1,703	534	3,368	6,986	783	17,083
	장출혈성대장균감염증	-	-	-	-	-	431	246	677
	A형간염	-	-	-	-	-	-	7,585	7,585
제2군	백일해	75,941	75,002	18,187	8,381	444	186	390	178,531
	파상풍	0	0	6	53	12	109	72	252
	홍역	67,901	11,6435	48,877	26,075	13,327	56,061	266	328,942
	유행성이하선염	25,314	32,945	15,186	10,483	13,084	28,099	36,747	161,858
	풍진	-	-	-	-	-	413	142	555
	일본뇌염	11,763	14,587	862	1,641	18	33	63	28,967
	수두	-	-	-	-	-	81,291	125,773	207,064
제3군	말라리아	-	8,497	35,689	0	9,765	18,576	3,585	76,112
	한센병	--	1,891	10,952	2,881	1,166	423	25	17,338
	성홍열	19	37	170	1,808	1,210	977	5,158	9,360
	수막구균성수막염	116	157	36	81	40	114	29	457
	레지오넬라증	-	-	-	-	-	106	104	210
	비브리오패혈증	-	-	-	-	-	536	244	780
	발진열	-	0	4	4	136	378	137	659
	쯔쯔가무시증	-	-	-	-	3,534	42,761	29,791	76,086
	렙토스피라증	-	-	-	596	432	1,193	193	2,414
	브루셀라증	-	-	-	-	-	620	83	703
	공수병	-	268	28	2	1	5	0	304
	신증후군출혈열	-	-	349	450	1,230	3,683	1,734	7,446
	매독(1기,2기,선천성)	-	-	-	-	-	-	2,551	2,551
	크로이츠펠트-야콥병	-	-	-	-	-	-	108	108
제4군	뎅기열	-	-	-	-	-	321	598	919
	큐열	-	-	-	-	-	51	42	93
	웨스트나일열	-	-	-	-	-	-	1	1
	라임병	-	-	-	-	-	-	16	16
	유비저	-	-	-	-	-	-	3	3
	치쿤구니야열	-	-	-	-	-	-	2	2
	중증열성혈소판감소증후군(SFTS)	-	-	-	-	-	-	36	36

Data Accessibility

감염병웹통계(http://is.cdc.go.kr)에서는 전수감시 대상 54종 감염병에 대한 지역별, 성별, 연령별, 감염지역별, 기간별, 전년대비 주간 발생현황 등 실시간 기간 검색이

가능하다. 검색된 통계를 표 또는 다양한 그래프로 볼 수 있으며, 또한 이러한 자료들은 엑셀로 다운로드하여 사용이 가능하다.

Key findings (and publications)

법정감염병 78종 중 24종에 대해서는 표본감시체계로 운영하면서 감염병 발생현황 및 추이를 파악하여 유행을 예측하고 있다. 신고자료는 환자개별 사례가 아닌 신고기준에 합당한 의사환자 수이다. 그러므로 대상 감염병의 발생수를 파악하기 어려운 단점이 있다.

이러한 표본감시 자료를 이용하여 2010년 '국내 인플루엔자 환자 및 중증 폐렴으로 인한 입원환자 발생규모 추정'에 관한 연구를 실시하였다. 이 연구의 주요 내용은 인플루엔자 외래환자 및 중증폐렴 입원환자의 발생규모를 추정하는 것이다. 인플루엔자 환자 발생규모 추정을 위한 모형개발을 위해 인플루엔자 표본감시기관에서 보고한 자료(2005년~2010년, 지역별 총 진료인원, 총 의사환자 수, 의사환자분율, 연령별 환자수)와 전국 의료기관에서 심사평가원에 인플루엔자 상병코드 J09-J11로 청구한 자료, 총 외래진료인원과 내원일수)를 바탕으로 그림 1과 같은 방법으로 추정모형을 개발하였다[4].

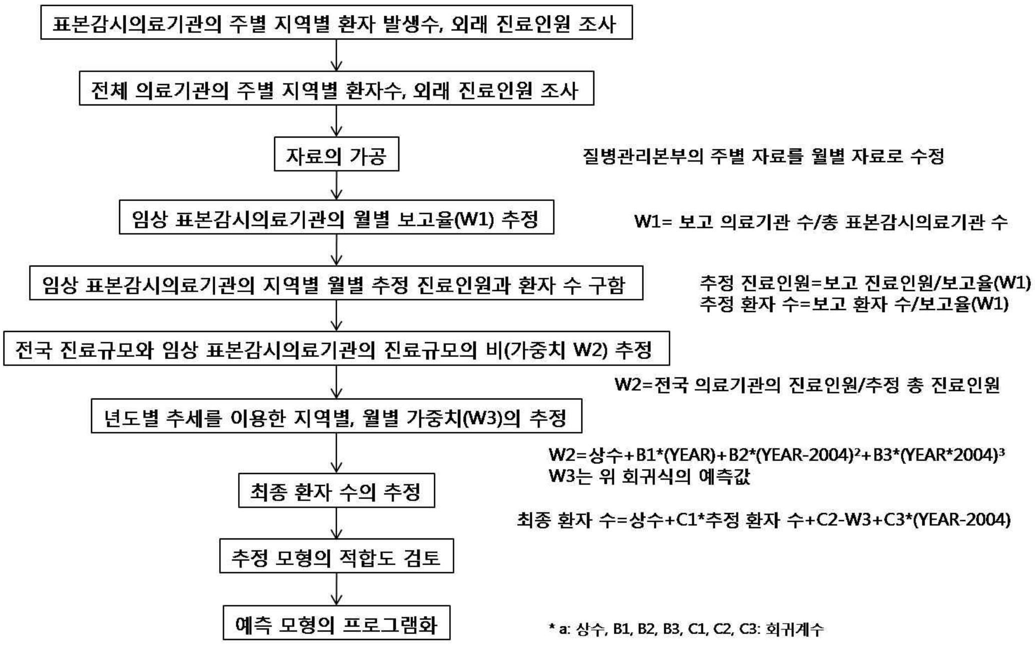

그림 1 인플루엔자 환자 발생규모 추정 모형개발 과정

이 과정으로 인플루엔자 환자발생규모를 추정할 수 있는 수식을 만들고, 이 수식을 이용해서 2009년 11월 서울시 인플루엔자 환자수를 계산하였는데 320,567건으로 건강보험심사평가원 진료비 청구건수와 비교해서 64명의 차이를 보였다.

최종 환자수 = 상수 + C1*(추정 환자수*W3) + C2*(YEAR-2004)

Strengths and weaknesses

법정감염병 정보수집은 최신 진단법, 역학적 특성 등을 반영하여 「감염병 진단·신고기준」매뉴얼을 제작하고 의료기관에서 사용함으로써 신고기준이 표준화되어 있다. 한편 우리나라 법정감염병 신고 및 보고 체계의 문제점은 의사의 신고에만 의존하고 있어 신고율은 낮은 형편이다. 따라서 감염병 환자수가 정확하게 수집되지 않은 감염병에 대해서는 실제 환자수를 분석 또는 추정해야한다. 질병관리본부에서는 의료기관을 대상으로 감염병신고 방법에 대한 홍보활동도 실시하고 있지만 효율적인 법정감염병 감시를 위해 신고수단뿐만 아니라 다른 다각적 방법 개발 하려고 계획하고 있다.

<References>

1. Korea Ministry of Government Legislation. Infectious Disease Control and Prevention Act.
2. Korea Ministry of Government Legislation. Infectious Disease Control and Prevention Act.
3. Korea Centers for Disease Control & Prevention. Infectious Diseases Surveillance Yearbook, 2013.
4. Korea Centers for Disease Control & Prevention. Modeling the national pandemic influenza cases and inpatients of pneumonia in Korea; 2011.
